# Editorial: Functional materials and device design in perovskite solar cells

**DOI:** 10.3389/fchem.2026.1878114

**Published:** 2026-06-10

**Authors:** Yang Wang

**Affiliations:** Strait Laboratory of Flexible Electronics (SLoFE), Fujian Key Laboratory of Flexible Electronics, Strait Institute of Flexible Electronics (SIFE, Future Technologies), Fujian Normal University, Fuzhou, Fujian, China

**Keywords:** advanced photovotaic technology, device engineering, functional materials, organic solar cell, perovskite solar cell

Over the past years, perovskite solar cells (PSCs) have emerged as a promising photovoltaic technology, driven by the unique properties of perovskite materials—including tunable band gaps, high absorption coefficients, and long carrier diffusion lengths—attracting significant interest from both fundamental research and industry. Through extensive efforts in functional material design and device engineering, the certified power conversion efficiency of PSCs has now surpassed 26%. To accelerate their commercialization, however, current efforts must focus on resolving stability issues, which call for further development of novel multifunctional materials, enhanced interfacial properties, stabilized perovskite active layers, and optimized device architectures. We are deeply honored to have compiled this Research Topic titled “*Functional Materials and Device Design in Perovskite Solar Cells*” which clearly captures the full contemporary scope of this research field, both from material design and device engineering. This Research Topic features four invited articles covering the latest progress in the field of next-generation photovoltaic including organic solar cells.

The first paper from Vishnupriya and Sathya presents an innovative dual-absorber solar cell architecture incorporating MAGeI_3_ and CsSnI_3_ layers, designed to surpass the efficiency limits of conventional single-junction cells. Using SCAPS-1D simulations, optimal thicknesses for both materials were identified, yielding a maximum efficiency substantially higher than previously reported for analogous configurations. Under varying defect densities, the optimized parameters produced a strong open-circuit voltage, high short-circuit current density, and an excellent fill factor, resulting in a notable overall efficiency even at moderate defect levels. This performance enhancement stems from the complementary bandgaps of the two materials, which boost light absorption and improve charge carrier dynamics. The findings of this study imply that the dual-absorber configuration has significant potential to drive progress in solar cell technology and therefore calls for further experimental verification.

The second article is a review paper from Tian and Zhang, summarizing recent progress in SAMs for PVSCs, with a focus on three key aspects: anchoring groups and interface engineering, electronic structure modulation and band alignment, and stability optimization. The review highlights the dual role of SAMs in passivating defects and enhancing crystallinity, while also enabling precise energy level tuning for more efficient hole extraction. The review also discusses co-adsorbed SAM strategies that improve device resilience against thermal and moisture stress. Collectively, SAMs offer a promising route to overcoming efficiency and stability bottlenecks in PVSCs, advancing their commercial prospects. Future efforts should target long-term environmental stability and scalable SAM deposition techniques for industrial application.

The third article from Yu et al. introduces 3,3′-diethyl-oxacarbo-cyanine iodide (DiOC2(3)) and multifunctional groups (C=N, C=C, C-O-C, C-N) into perovskite precursor solutions to simultaneously passivate deep level defects and reduce recombination centers. The multifunctional groups in DiOC2(3) coordinate with free Pb^2+^ at symmetric sites, thereby passivating lead vacancy defects, effectively suppressing nonradiative recombination, and maintaining good stability. As a result, the power conversion efficiency (PCE) of an inverted 1.68 eV wide-bandgap (WBG) perovskite solar cell increases from 18.51% to 21.50%, with a Voc loss of only 0.487 V. Moreover, the unencapsulated device retains 95% of its initial PCE after 500 h of storage in a nitrogen environment at 25 °C.

The last article written by Theunissen et al. is a perspective paper focused on single-component organic solar cells. The article briefly surveys the current state of single-component organic solar cells fabricated from conjugated block copolymers (CBCs), discussing the challenges associated with their characterization, emphasizing the critical role of chemical precision in these molecular architectures, offering several practical recommendations, and examining how these factors may shape the future development of the field.

This Research Topic brings together original studies that cover the current aspects of research in the field of advanced photovoltaic technologies including perovskite solar cells and organic solar cells. We extend our sincere gratitude to all contributing authors for their outstanding work and for sharing the latest advances within this Research Topic. We also sincerely thank all reviewers for their rigorous and timely evaluation of the manuscripts, despite their busy schedules. Finally, if the content of this Research Topic proves to be both practical and inspiring—not only to experts but also to young researchers entering this exciting field—we will regard our efforts as truly successful.

